# Life loaded with threat and vulnerability: a qualitative inquiry into the experiences of HIV negative married women in serodiscordant heterosexual relationships

**DOI:** 10.1186/s12905-021-01546-4

**Published:** 2021-12-07

**Authors:** Mona Larki, Narjes Bahri, Robab Latifnejad Roudsari

**Affiliations:** 1 Student Research Committee, Mashhad University of Medical Sciences, Iran Mashhad,; 2 Department of Midwifery, School of Medicine, Social Development and Health Promotion Research Center, Gonabad University of Medical Sciences, Gonabad, Iran; 3 Nursing and Midwifery Care Research Center, Mashhad University of Medical Sciences, Mashhad, Iran; 4 Department of Midwifery, School of Nursing and Midwifery, Mashhad University of Medical Sciences, Mashhad, Iran

**Keywords:** HIV, AIDS, HIV serodiscordant, Violence, Gender-based violence

## Abstract

**Background:**

Violence against women is a major, complex, multidimensional and widespread public health concern worldwide. The current qualitative study was conducted to understand the experience of violence among HIV negative married women in heterosexual serodiscordant relationships.

**Methods:**

A qualitative description (QD) was conducted from October 2018 to January 2020 in Mashhad, Iran. The participants were 15 HIV negative women, who married and lived with HIV positive men, through purposive sampling method. The data were collected using semi-structured interviews. Data analyzed using conventional content analysis adopted by Graneheim and Lundman.

**Results:**

The main overarching theme emerged entitled: life loaded with threat and vulnerability. This theme consisted of four categories of self-directed violence, intimate partner violence, cultural violence and structural violence. The violence began soon after awareness of husband's infection with acts such as suicide attempts and a sense of abhorrence for living with an infected person, and continued with confrontation with various types of violence in the family and society, which put women in constant threat and vulnerability.

**Conclusions:**

This study provided an insight into different aspects of violence in Iranian women in HIV serodiscordant relationships. Considering the role of men in the occurrence of violence, policymakers must create and execute family-centered interventions to address attitudes and behaviors that lead to marital conflicts and spousal abuse in order to prevent violence. Health care professionals should also be trained to screen women for violence and refer those who require care to specialists to reduce vulnerability.

**Supplementary Information:**

The online version contains supplementary material available at 10.1186/s12905-021-01546-4.

## Background

The Joint United Nations Program on HIV/AIDS 2019 (UNAIDS) announced that, more than 37.9 million individuals currently live with HIV worldwide [1]. It is the second most common cause of mortality among communicable diseases [2], and will become the first cause of death by 2020 [2]. According to the latest UNAIDS spectrum and analysis [15], 59, 000 [95% confidence interval (CI) 33,000–130,000] people in Iran are infected with HIV in 2019, with approximately 4100 (95% CI 1200–12 000) new infections and 2500 (95% CI 1200–5600) AIDS-related death occurring each year [3]. Based on data from Iranian national HIV registry, 38,966 people were infected with HIV by the end of 2018. The majority of the patients were men (83%) between the ages of 16 and 40 (67.6%). Also, there were 15,845 HIV-positive people who died of any cause [4].

Violence and the fear of violence have been identified as an important key factor for vulnerability to HIV infection for women of different countries [5]. Violence against women is a critical, complex, multidimensional and widespread public health concern [6]. Self-directed violence, interpersonal violence, and collective violence are the three broad types of violence included in the World Report on Violence and Health (WRVH) by WHO, according to whom performs the violent act. Despite not being generally accepted, the WRVH also gives a typology of violence that can be useful for understanding the contexts in which violence occurs and the relationships between different types of violence. This typology distinguishes four types of violence: physical, sexual, and psychological assaults, as well as deprivation [7]. Based on the UN domestic and sexual violence, sexual harassment, and psychological types of harassment are the most common types of Violence Against Women VAW [8]. This phenomenon embedded in cultural, social and family patterns [6].

Recent statistics by the World Health Organization showed that one in every three women is vulnerable to physical and sexual violence and UN (United Nations) added that only 40% cases of violence seek for any sort of help [9]. According to a meta-analysis conducted in 2016 in Iran, including 31 studies on domestic violence (DV) against women (from 2000 to 2014), it was found that DV prevalence in Iran is estimated to be 66 percent. The prevalence rate of DV was 70% in the Eastern region, 75% in the West, 62% in the North, 70% in the South, and 59% in the Central region [10]. In another study in 2019 in South Western Iran, the overall rate of DV was 54.5% and the emotional, physical, and sexual domestic violence were found to be prevalent in 52.0%, 18.2%, and 14.0% of cases, respectively [11].

One of the key populations for prevention of HIV are serodiscordant couples [12]. HIV serodiscordant relationship refers to a mixed-status and situation, where one of the partners is HIV positive, while the other is HIV negative [13]. Among people with HIV who are in stable relationships, up to 50% are in serodiscordant relationships [14]. HIV serodiscordant relationships, increase the odds of HIV negative partner being infected by HIV by 8–26% yearly compared to the other couples [15]. When partners make aware of their HIV status together in serodiscordant relationships often face major challenges including gender-based violence, lack of sexual desire, divorce and abandonment, self-blaming, the stress of possible transmission, financial pressures, HIV-related stigma, family breakdown, desertion and isolation; all of which may have a negative influence on their relationships [16–20]. A study conducted on serodiscordant couples, indicated that exposure to intimate partner violence differed significantly between men (28.6%) and women (89.3%) [21]. To the best of our knowledge, no study has reported the epidemic of serodiscordant couples and the prevalence of intimate partner violence among them in Iran. Mashaphu [21] argued that there is a high level of intimate partner violence in HIV serodiscordant couples, therefore, intervention programs should address gender-based violence and inequity among heterosexual couples. Patel [22] suggested that counseling programs in relation to intimate partner violence may be important in preventing the risk of HIV transmission to HIV-negative wives.

The issue of violence, which is occurred within a social context could be influenced by gender norms, interpersonal communications and sexual stereotypes. Many have debated that women’s experiences of violence could not be examined with traditional quantitative approaches, regardless of its context [23, 24]. Qualitative research methods have enhanced in-depth insight into the subjective experience of violence and provide a better understanding of the manifest and latent meanings embedded within the context [25]. Besides, qualitative research is a very useful and rigorous approach for investigating sensitive topics such as HIV and violence [26]. It seems that conducting research about women’s experiences of violence is essential for the reason of identification, prevention, control and elimination of violence and also to provide required support services for female victims of violence [27]. More studies which have been conducted in this context in Iran have focused on HIV positive women and there is no evidence to explain the experiences of violence in Iranian HIV-negative women, who live with HIV-positive husbands i.e., HIV serodiscordant couples. Hence, the current qualitative study was designed and conducted with the purpose of understanding the experience of violence among HIV negative women in serodiscordant heterosexual relationships.

## Material and methods

### Design

We used a qualitative description (QD). Thorne et al. [28] introduced QD as a collection of procedures for conceptual orientation, sampling, data construction, analysis, and reporting that health scientists may utilize as an interpretative descriptive method to generate knowledge about the human health and disease. QD is a systematic approach to doing qualitative inquiry because of the philosophical, ontological, epistemological, and methodological underpinnings it. Researchers who carry such investigations have the least pre-existing theoretical and philosophical commitments in terms of theoretical orientation [29, 30].

### Participants

For the purpose of this study, the Clinic of Behavioral Disease Counseling was chosen as the study setting. The reasons for the selection of this clinic were that it was located in the central part of Mashhad, Iran and all patients with HIV and their wives referred to it to receive their health and counseling services and their medical records were available too. The study was conducted from October 2018 to January 2020 (16-month period). The participants were 15 HIV-negative married women who lived with HIV-positive men. The inclusion criteria for the study were women with serdiscordance relationship, the ability to talk and express emotions and feelings, and the desire to participate in the study. Participant with unwillingness to continue the interview were excluded from the study.

### Sampling

The purposive sampling method was used to draw the sample for the study. To recruit participants, one of the health care providers in the clinic contacted the eligible members by phone call and explained the research objectives to them. Then, a convenient time was set for interviews with the individuals, who agreed to take part in the study.

### Interview procedures

The method used to data collection was semi-structured interviews. The interviews were semi structured and carried out individually and face to face. All interviews were conducted in a room at the Clinic of Behavioral Disease Counseling. Due to the sensitivity of the phenomenon under investigation and in order to achieve a greater insight into the experiences of the participants, all interviews were conducted by the first researcher, who is a reproductive health researcher with previous experience of interviewing females with high-risk sexual behavior in prison. An interview guide was used to conduct face-to-face interviews (see Additional file 1: Appendix). The interviews started with an open-ended and general question: Tell me more about the relationship with your husband? Then continued with main questions: what experiences do you have of being victim of violence? Probing questions were also used during the interviews: can you give me an example? Could you please explain more about that? Also, during interview process, researcher considered non-verbal cues such as body language. The interview continued until saturation was achieved and no further concepts were detectable in the participants' experiences. One interview was conducted with each participant. The length of Interviews was between 50 and 70 min.

### Ethical considerations

All participants in this research volunteered to take part in the study after being explained the objectives, the benefits, confidentiality and anonymity of information they were going to give. Considering that the issue under investigation was very sensitive to achieve a great insight, all interviews were conducted by one researcher (ML). All participants were given an informed consent form in Persian which was the native language of the participants and the researchers, which they read and signed. In addition to the verbal consent, written consent was also obtained from the participants. Also, it was emphasized to all women that they can withdraw from the study at any time without any prejudice. If the participants were not comfortable to answer any question, they were not forced to give response. All interviews were conducted in the participant’s native language (Persian). It was explicitly stated to the participants that if they decline to participate in the study, it would not influence their provision of care” in order to eliminate “fear, unconscious coercion, and secrecy” [31]. To achieve greater intimacy, the first author spent considerable time at the Infectious Disease Clinic with participants before and after each interview, answering their questions, and talking to women about various issues other than HIV. The researcher gave an opportunity to participants to ask any questions about the subject under study after the interview completion. Before the end of the interview, each participant was asked for agreement to be contacted again in the future, if necessary, in relation to this study. All the participants agreed for it and gave their phone number to the researcher. It should be noted that the researchers did not contact any of the women for a subsequent interview.

At the end of the interview, a gift was given to the participants for compensation. All the information collected from participants was kept confidential and instead of using their names, they were given unique numbers to be used in analysis. If necessary, the participants were referred to the psychologist of Infectious Disease Clinic because we were not qualified to offer counseling and therapy in the field of psychiatry. Ethical approval for the current study was obtained from ethical committee of Mashhad University of Medical Science (Code of Ethics. IR.MUMS.NURSE.REC.1397.022).

### Data analysis

All interviews were audio-recorded, transcribed verbatim and entered into MAXQDA version 12 that was developed and distributed by VERBI Software based in Berlin, Germany. Data analysis began immediately after the first interview. The analysis process was first conducted in Persian and then translated into English. We used the Graneheim and Lundman’s method [32] to analyze the data. At first the text of the interviews by two coders (ML and NB) was read several times to obtain a general understanding and sense of the whole of their content. Then the text of each interview was divided into meaningful units as words, phrases, sentences, and paragraphs. Thereafter the meaning units were condensed and labeled with code. Then the meaning of codes was compared in terms of similarities and differences, and the related codes with similar meaning were put in the initial categories. Finally, with the advancement of data analysis, the initial categories or subcategories were developed and categories formed, from which an overarching theme was emerged. As a result, the data-driven categories evolved progressively and inductively from the text data (Table 2).

### Trustworthiness

In this study, credibility was established through interviews and including appropriate number of participants using maximum variation strategy. Also, the field notes, which contained the researcher’s thoughts and feelings, impressions, as well as the interpretation of interviewees’ non-verbal cues and body language enhanced credibility. Indeed, the field notes were written by the first author after each interview to identify any differences that she noticed, as well as to set aside her own impressions, emotions and assumptions about the research focus and in order to keep an open mind in communication instead of being judgmental. Also, field notes prompted the development of the next set of questions to be asked of other participants. It is noteworthy that non-verbal communications have long been recognized as a valuable source of information and a useful supplement to the inquiry of human verbal behavior. Actually, what a person does not say may often reveal by body language more than what he or she says. Qualitative researchers can obtain useful information from participants' silence [33]. As an instance in participant 4, her purposeful silence was a symbol of her approval of her words.

For dependability, the researcher gave data to an independent coder who was skilled in the field of qualitative research to do an independent examination of the data and confirm them. To ensure confirmability supervisor review of findings, interpretations, and conclusions of the study greatly increased the confirmability of this study; so, conducting an audit trial was possible. For transferability, clear and thick description of culture, context, the method of participant selection, and characteristics of participants as well as the process of the data analysis was provided.

## Results

### Participant characteristics

The participants were 15 married women. The ages of the respondents ranged from 30 to 60. Exception of one couple, all couples had children. Four of the couples (26.6%) had one child, while two couples had four. The age of the children ranged from under seven to over thirty-four. The majority of women were in their childbearing age, when they discovered their status. Most of the participants completed lower secondary school education and three had a diploma. The majority of women chose not to disclose their status to their children. Only one participant informed her children and one informed her entire family. The characteristics of participants are presented in Table 1.

**Table 1:** Socio-demographic profile of women involved in the interviews (n = 15)

Characteristics	Mean (range or percentage)
Age (years)	38.8 (30–60)
Education	
Primary	3 (20%)
Intermediate	7 (46.6%)
High school	2 (13.3%)
Diploma	3 (20%)
Duration of marriage (years)	16.06 (5–35)
Length of being aware of husband's illness (years)	5.4 (1–12)
Occupation	
Housewife	12 (80%)
Worker	1 (6.66%)
Seller	1 (6.66%)
Tailor	1 (6.66%)
Husband's occupation	
Temporary employees	8 (53.33)
Owned private businesses	3 (20%)
Unemployed	3 (20%)
Employed	1 (6.66%)
Economic status	
Poor	10 (66.66%)
Relatively appropriate	4 (26.66)
Good	1 (6.66%)
Number of children	2 (0–4)

**Fig. 1:**
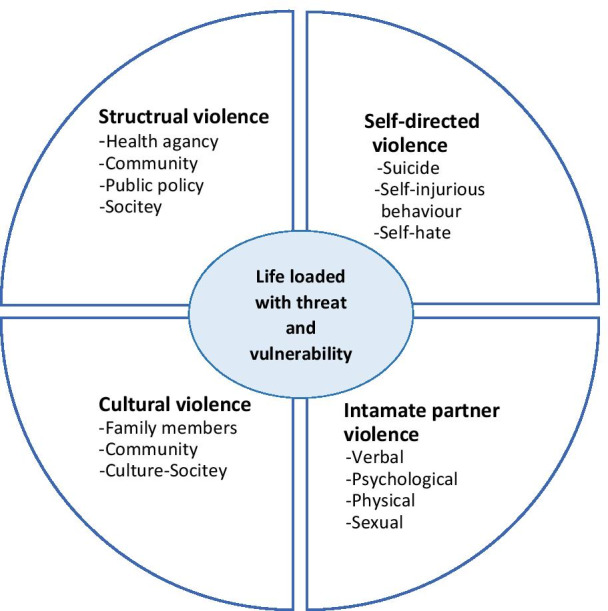
Types of violence in HIV negative women in serodiscordant heterosexual relationships

The results of the analysis included 96 meaning units, 12 subcategories, four categories and one theme. Examples of the inductive process in the content analysis are shown in Table 2. The main overarching theme emerged entitled: life loaded with threat and vulnerability. It consisted of four categories of self-directed violence, intimate partner violence, cultural violence and structural violence (Table 3) (Fig. 1).

**Table 2:** Examples of the steps in the analysis process

Quotes of married women in serodiscordant heterosexual relationships	Meaning unite	Condense meaning unit	Code	Sub category
I believe that men in cultural context of Iran have been granted power over women	Men have been granted power over women in Iranian culture	More power of men than women	Power imbalance	Gender stereotypes and patriarchal culture
According to the relatives and parents, the man is the head of the family and must be considered regardless of status	Man is the head of the family in any situation, according to relatives	Man is the head of the family based on family members' perception	Predominate role of men in the society	
He starts to think and behave violently, after he marries with me, because he thinks that owns me	Men believe that after marriage, they own women and can use violence against women	Becoming the owner of women after marriage	Dominance of masculinity	
I believe that if they [men] passed the marriage ceremony, they have the authority to do whatever they want with their wives	After marriage, men can do whatever they want with their wives	The authority to do whatever men want with wives	Full authority of men over women	
He has power over me and could control me… So, I think I am just an instrument in his hands… Even though I am HIV-negative now	He has power over me and can control me. I'm like an instrument	He has power and control over me	Excessive control by the husband	Psychological aggression
Ever since he infected with AIDS, if my phone is busy when he calls, he will suspect that I am in another relationship	If my phone is busy, he may suspect that I am in another relationship because he has AIDS	He is suspicious that I am in another relationship	Being accused of betraying by husband	
My husband always ignores me in front of my parents and kids that make me feel ashamed. Maybe it's because of the side effects of AIDS-related drugs…	My husband ignores me in front of my parents and kids, which makes me ashamed	My husband ignores me in front of my parents and kids	Being ignored by husband	
He assaults not only me, but also my relatives, particularly my parents, whom he believes defend me when I have got problems	He insults me and my family because he thinks my family supports me	My husband insults me and my family	Verbal misconduct of husband toward my family	Verbal harassment
He often shouts at me, even in front of the kids. I feel really ashamed of shouts my husband perpetrates on me in the face of our children and other family members	He often shouts at me, even in front of others	shouts at me, in front of others	Verbal misconduct of husband toward me

**Table 3:** Theme, categories and subcategories emerged from the data analysis

Subcategories	Categories	Theme
Suicidal ideation	Self-directed violence	Life loaded with threat and vulnerability
Self-Injurious behavior		
Self-hate		
Verbal harassment	Intimate partner violence	
Psychological aggression		
Physical harm		
Sexual assault		
Social marginalization	Cultural violence	
Gender stereotypes and patriarchal culture		
Deprivation of the right to economic participation	Structural violence	
The lack of legal protection		
Health disparities

### Main theme

The main theme that derived from the analysis of data was "life loaded with threat and vulnerability." The violence started almost immediately after the women became informed of their husband's disease, with destructive actions and feelings. Then, they encountered various forms of violence, including physical, psychological, verbal, and sexual, while living with their abusive husband. They were also threatened in the community due to the complexity of AIDS, and they were experienced to violence that was embedded in the society's culture and structure. Women's experiences showed that they had a life that is constantly full of threats and vulnerabilities.

#### Category 1: self-directed violence

A topic raised by study participants was self-directed violence. Women sometimes experienced self-injuries activities in the event of confronting with illness of husband or reactions of relatives.

##### 1-1: Suicidal ideation

Some women disclosed that their husbands engaged in high-risk sexual behavior such as occasional extra-marital relationships, which resulted in having suicidal thoughts. In this regard, one participant said*: "When I was pregnant, I understood he betrayed me. I was suspicious of my husband’s behaviors and I recently found a condom in his pocket. I was afraid that my surroundings do not accept me. Most of the times I think what should I do? Because of this, I tend to commit suicide for fear of getting AIDS" (P2. 38 Y).*

##### 1-2: Self-injurious behavior

Some women disclosed that their husbands engaged in high-risk sexual behavior such as occasional extra-marital relationships, which resulted in having suicidal thoughts. One female said that, *"My husband is not concerned about living expenses, for instance, if I want to buy anything he never agrees with. He sells home appliances and doesn’t pay any attention to the kids. I take care of the children and always seek to give them comfort, but is it just my responsibility? In this condition, I do not want to eat and I like to hit my head against the wall" (P2. 38Y).*

##### 1-3: Self-hate

The women interviewed mentioned that living with an HIV-infected husband had consequences for their psychological system. Most of the participants stated that their activities were restricted by their husbands. It led to their negative feelings about themselves. Only one participant stated that her activities are not limited by her husband:* "I can't do anything without my husband's permission. I wish I could work outside and I could be financially independent. But my husband prevents me from working, he does’not financially support me at all. The dependency makes me more vulnerable to my husband’s acts of violence … I sometimes hate myself for not getting divorced" (Touching her face) (P11. 40 Y).*

#### Category 2: intimate partner violence

Women in their serodiscordant relationships with a partner are usually victims of a combination of violence including verbal harassment, psychological aggression, sexual assault and physical harm.

##### 2-1: Verbal harassment

Women experienced verbal violence in a variety of forms such as scurrility and screaming*.* One woman said that*: "When we speak to one another, he uses bad word. He begins to scream and cannot control himself at all. When he comes home, he stars constantly ordering me to do something with a high voice. He feels pride doing it" (P 2. 38Y).*

##### 2-2: Psychological aggression

Psychological violence includes controlling daily commutes and phone calls, scepticism, humiliating a woman or her family or ignoring her and her family members. The findings showed that almost the majority of the participants in the study mentioned psychological violence by their husband. One woman expressed that, *"my husband is very suspicious. He constantly checks my mobile phone, even though my phone is always at home. He consistently says that women are worthless. When my husband says it, my confidence is going down" (P5. 30Y).*

##### 2-3: Sexual assault

A few of serodiscordant women talked about sexual assault. Sexual violence victims who shared their stories have reported low or very low levels of this type of violence. They indicated having constant arguments and disputes towards sexual issues. One woman has mentioned forced unprotected sex with her husband: "*I strongly believe condom continue to be used before a cure is found for HIV. But he doesn’t want to use the condom. He said, I don’t like using a condom. In this condition I cannot protect myself, because my husband doesn’t put on a condom. I am forced to give him sex because I fear that he embarrasses my children" (P11. 40Y)*.

##### 1-4: Physical harm

The women interviewed stated that their husbands beat them in various ways at home, the most common of which was hand beating. But in some cases, this was done by pulling their hair or banging their head against the wall, as well as pushing and throwing them. The effects of physical assaults were usually more expressed than other types of violence.. *One female stated: "he beat me up in front of kids. He used physical force such as hands and feet for choking and shoving me. I begged him to stop, but he said I like killing you and the kids" (P7. 37 Y)*.

#### Category3: cultural violence

Cultural beliefs could play an important role for HIV / AIDS patients and their families in adapting, coping with the disease and violence toward them and their families. Participants said that when relatives became informed that their spouse is HIV positive; they reject them from the family. Also, it was resulted in labeling and stigmatization in the community.

##### 3-1: Social marginalization

Serodiscordant women chose not to disclose their status to their family and friends, as they found that women who disclosed their status were rejected by their relatives. The following quote explain this point: *"My family' behavior has changed toward me. I am seronegative, but some people, who see me at the clinic with my HIV positive husband, they think that I am positive too. My family used to gather and eat together, but when they know that my husband is HIV positive, left their habit. In our society when people hear that such a woman has a HIV positive husband it is a very shameful condition. I think, this is an example of overt violence. I say to my husband that I am really suffering from this problem" (silent) (P 4. 45 Y).*

##### 3-2: Gender stereotypes and patriarchal culture

The women explained that due to the interference of families, especially the husband's family they have to face several problems. A participant specifically mentioned about her mother-in-law interference: *“She believes in women's complete obedience to men and inherent aggression against them. My husband spends most of his time at her home and used to listen to his mother advice, who is not a right person for consultation. For example, she said to my husband you have power and you can transfer it (HIV) to your wife and you can infect her. My mother- in-low and I is always arguing together" (P 6.34 Y)*.

#### Category4: structural violence

Structural violence, which reveals inequality in the distribution of jobs and resources among people living with HIV / AIDS, was one of the issues that serodiscordant women experienced due to living with HIV positive men. They confronted with deprivation from economic participation, the lack of legal protection and also health disparity.

##### 4-1: Deprivation of the right to economic participation

Participants stated that their husbands lost their job and sources of income when their employer was informed of their status. One woman mentioned *"my husband decided to disclose his status to the company, but they told her that they couldn’t give her a job. He is unemployed. He afraid to go to other factories to look for a job, you know, because he feels that he will be approached the same. Several times I went to look for a job for him, but did not approached well. In this situation he’s become more nervous with me and the kids"(P 8. 39Y).*

##### 4-2: The lack of legal protection

From the interviews conducted with the women, it was found that the majority of the women still has not reported the violence to the legal organization because they thought that they would not be supported by the legal system. One woman mentioned: *"The law does not give sufficient protection for women in instances of violence. Men also know it. Once I went to court to divorce, but the judge did not pay any attention to me and said, you are a mother, you must endure" (P 12.36 Y)*.

##### 4-3: Health disparities

The majority of participants in this study had strong relationships with their health care providers. But a few participants referred to the bad experiences from the healthcare providers in hospital. A woman who had a good relationship with her husband in this relation said: *"When he (my husband) got sick, I strongly believed that, it is my responsibility to accompany her for going to the hospital without shame, but in the hospital where we went for care, one of the care providers loudly said that he is HIV positive. Also, the nurse gave care to my husband with a violent behavior, you know, and when I protested him, he, with a bad tone, said, your husband is HIV positive and I should take care of myself. In this situation, I just cried and came out of the room. I felt so bad" (Lip biting) (P 13. 35 Y).*

## Discussion

The key findings that emerged from the study indicated the experience of various types of violence among HIV serodiscordant women. Violence, rooted in misconceptions about AIDS, began soon after the awareness of husband's infection with acts of suicide attempts and a sense of hatred for living with an infected person, and continued with confrontation with various types of violence in the family, and society. HIV is a phenomenon in serodiscordant relationships that involves both partners. It is associated with unique stressors. In a study a high rate (83.1%) of suicidal attempt was reported in HIV-positive people [34]. It is also, a stressful event in women's life that led to self-directed violence. In this study the major problem in females was psychological reactions in dealing with the issue of difference in serostatus. They bear the psychological pressure from their husband and the society, which often led to psychosocial problems as self-hate. Although in these cases violence often occurs by an intimate partner but it takes in different forms such as violence by a family member or relatives [35]. Part of the experience of violence is related to interacting with the husband. We categorized intimate partner violence to verbal, sexual, psychological and physical. In general, women in their relationships with a partner are usually victims of a combination of violence. So, it seems difficult to separate the different dimensions of violence [36]. In this study women explained that they had low power and authority for decision making in their life issues. Psychological and verbal violence included behaviors that intended to undermine women’s confidence and self-esteem. Some of the verbal violence included the use of words such as foolish, fat and silly [37].

Psychological violence can be subtle and there is no clear definition for it [38]. In fact, it may be more effective than physical violence on women’s emotional status. Also, UNESCO states that psychological abuse has not been given proper attention [39]. Sexual violence was presented by a small number of women, since, it seems that women based on cultural norms and religious beliefs, felt obligated to respond to their husband's sexual desires under any circumstances. They attribute this issue to the self-esteem of the spouse and to the submissive and obedient and dutiful role of women. This type of violence would affect living opportunities and social status of women. Also, fewer statements were attributed to women's self-censorship and shame in this study. Managing emotional and sexual intimacy can be challenging in HIV serodiscordant relationships because of concerns about HIV transmission, the burden of initiating and maintaining safer sex, and the health status of the affected partner [40]. This study showed that serodiscordant couples have numerous challenges about sexual relationship. Serodiscordant women indicated that there is a lack of regular condom use in some men in sexual relationships. Men consider having sex without any protection as a right for themselves. In this condition, men face women's objection, and this itself leads to sexual and psychological violence against women. This finding is congruent with the results of a study conducted in Uganda in which women's fear of HIV transmission and frequent demand for using condom by the male partner played an important role in the sexual violence [41]. One study reported that HIV-infected men deliberately infect their partners with HIV infection [42]. McDonald et al. [43] declared that condom in serodiscordant relationships is as a reminder of serodiscordance, which would reduce the motivation for use. This issue can adversely affect women's sexual desire and may lead to increased psychosexual problems that often escalate violence [44]. For HIV uninfected women, who are in an abusive relationship with coercive sexual intercourse, there is a reduced ability to negotiate for safe sex and as a consequence their chance for getting HIV is increased [45]. In the study by Lotfi et al. [46] in Iran exploring perceptions of women at risk of HIV/AIDS regarding gender norms and gendered power relation, it was found that women perceived feelings of fear and occurrence of violence as an obstacle to convince their husbands to use condom.

A study by Smith and colleagues describes women's experiences of physical, emotional and verbal violence. Women were burned with cigarettes, thrown to the wall and were attacked in different ways. Some of them experienced emotional intimidations along with physical threats with a weapon. Their partners insulted them verball. Besides, they controlled women and violated their right of freedom, including having the right of financial independence [47]. The results of another study showed sexual violence in the form of coercion in sex, intercourse with the purpose of causing pain and verbal acts of humiliation [48].

Part of the experience of violence is related to interactions in society. It is a type of violence that is not visible but its effects can be felt. In general, women in our study not only lacked social support, but were also subjected to violence by relatives. Social exclusion was occurred following disclosing of HIV by families and community; although, the structure, source and consistency of social support networks, play a significant and successful role in helping women with abusive partners [49]. The findings in our study about social marginalization are supported by other studies. In HIV serodiscordant relationships, the HIV-negative partner also confronts stigma and social challenges [50], because HIV/AIDS is a family disease, and when one member of the family has HIV/AIDS, its impact can be felt throughout the entire family [51]. So, couples often tend to hide the disease from community members in order to protect themselves from stigma and violence. Rispel et al. [52] in a qualitative study on serodiscordant couples concluded that the experiences of the stigma were common in these couples, including, expressing rumors and labeling an HIV-negative person as HIV-positive one. In some traditional societies, there is a negative attitude towards fulfilment of women's rights. Traditional beliefs that men have a right to control women and its acceptance by women has made women and girls vulnerable to violence and led to a greater gender discrimination in the communities [53]. The exploitation of female and the right of husbands to threaten their wives physically is rooted in a long patriarchal tradition: the tradition that men are the head of household and women have to obey them [54]. One study in Kolkata found that with the severity of the disease, a large number of patients loss their jobs, confronted with reduced family income and increased expenditure for care seeking, and face greater economic outcomes, reflected by selling assets [55]. Although all women have experienced violence in this study, but only one of the women reported it to the law enforcement authority. This is consistent with the findings of other studies and leads to non-authority figures of violence [41, 56]. In this study, women were neglected when they came to law enforcement agencies. Women considered this situation as violence against themselves. In one study it was reported that half of the participants in their sample of abused women were dissatisfied with the police response to their assailants [57]. It should be considered that violence is a social issue and requires attention in criminal justice systems [58].

WHO notes the establishment of international and national legal structures promoting gender equality and strengthening police and other criminal justice agencies' responses to violent cases [59]. In addition, women in our study also experienced violent behaviors by health care providers due to the protests to their discriminatory behaviors with the husband. A study in China on more than 1000 health care providers showed that they had stigmatizing attitudes towards HIV patients like perceived social norms in the general population [60]. Stigma from health care professionals is a worrying issue because it restricts access and use of services to couples [61]. HIV/AIDS in addition to affecting community health leads to socioeconomic problems for individuals, families, communities and governments of many countries [62].

### Study limitations

There is a possibility that sociocultural issues are barriers for disclosure of violence and may hinder women to reveal the violence so that this might influence the responses of the participants. This study was conducted on a population of women attending the Behavioral Disease Counseling Clinic, and other women who did not attend the clinic were not interviewed, therefore, the findings are representative of only women who attended clinics. Also, men's perspectives have not been explored in this study, as they are a significant complement to their wives' views.

### Strengths

Despite the aforementioned limitations, there are several strengths to this study. The studies conducted about HIV/AIDS has mainly focused on the HIV positive individuals rather than HIV negative ones [63], but we conducted the study by qualitative method to obtain negative HIV women's viewpoints, who were in HIV serodiscordant relationships. The focus of the interviews was not only on the intimate partner and domestic violence, instead other forms of violence were also considered to provide insight in various aspects of violence. Furthermore, the focus of the study on perspectives of Iranian women is actually a strength of the study, since to the best of our knowledge, there is no study about Iranian women with serodiscordant relationships.

### Recommendations

According to the findings of this study, it is recommended that HIV intervention programs should address gender-based violence among seroderodiscordant couples. It is argued that these findings can help policymakers in designing care plans and empowerment programs for HIV serodiscoedant couples. In addition, our findings indicate that there is a need to reform the rules of dealing with a spouse who has committed violence against women. In order to better understanding of the problem of violence against women there is a need for more research. Suggestions for further research include investigating the perceptions of HIV positive men towards violence, to examine the cultural issues affecting the violence against women. Also, understanding how HIV-negative women experience violence following disclosure of their HIV positive husband's illness needs more investigation in the future research. Given that some of the men in this study were involved in extramarital relationships, which is a risk factor itself for HIV/AIDS, studies should be conducted to understand how the dynamics that erode the marriage/trust could create environments where men are acquiring HIV outside of their relationship. Additionally, measuring the prevalence, causes and types of violence using quantitative methods in HIV serodiscordant couples is recommended for further research. Due to the fact that some healthy women with an HIV/AIDS husband are hesitant reluctant to attend health centers to receive care. It is suggested that the focus be on empowerment and social interventions outside of the HIV clinic that address some of the issues of social norms and cultural violence, also help these couples improve conflict resolution skills and rethink patriarchal thought and behavior patterns.

## Conclusions

This study provided an insight into different aspects of violence in Iranian women in serodiscordant HIV relationships. In summary, women experienced all types of violence. Traditional beliefs in the community and the lack of appropriate laws and services to deal with perpetrators of violence lead to continuing violence against women. According to the findings of this study, an effective response to violence against women should include improving women's empowerment and enhancing their awareness of prevention strategies, decreasing patriarchal culture, and strengthening accountability for survivors of violence through the approval of effective laws. Also, attitudes and behaviors that lead to marital conflicts and, eventually, spousal abuse should be addressed. Therefore, considering the role of men in the occurrence of violence, policymakers must create and execute family-centered interventions to prevent violence. Health care providers at HIV/AIDS counseling centers should be able to address topics related to the violence for HIV negative women in serodiscordant relationships. This requires that they to be trained for screening of women for violence and refer those who need care and therapy to the specialists.

## Supplementary Information

**Additional file 1.** Interview guide.

## Data Availability

On reasonable request, the datasets used and/or analyzed during the current study are available from the corresponding author.

## Abbreviations

UNAIDS: Joint United Nations Programme on HIV/AIDS
UNESCO: United Nations Educational, Scientific and Cultural Organization
