# Supplementary material for: Life loaded with threat and vulnerability: a qualitative inquiry into the experiences of HIV negative married women in serodiscordant heterosexual relationships
Source: BMC Womens Health. 2021 Dec 7;21:402. doi: 10.1186/s12905-021-01546-4 (PMC8650320; doi:10.1186/s12905-021-01546-4)
Supplement: Supplementary file 1 — Additional file 1. Interview guide. [file 12905_2021_1546_MOESM1_ESM.docx]

**Interview Guide**

- What are your views about violence in general?
- What experiences do you have of being victim of violence?
- What have been your general experiences with your husband? Tell me about your experience if you do not mind?
- I'd like to get a feel of how free you are in your daily life. Could you share some of your own personal experiences about this?
- Have you ever been physically hurt by your husband? (For e.g., kicked, slapped, pushed, hit etc.) Can you tell me more about your experiences with regards to this? Could you tell me about your own experiences about this?
- Has someone ever influenced your choices about birth control, pregnancy, or safe sex?
- While discussing these topics might be uncomfortable, it's important for me to know whether you've ever been pressured or forced to do sexual behaviors you didn't want to do or that made you feel terrified, embarrassed, or humiliated during your marriage.
- Do you think there is anything in the Persian culture that encourages violence?
- Is there anything else you would like to tell me about what we discussed today, or anything else you think should know?
